# Pre-Existing Diseases of Patients Increase Susceptibility to Hypoxemia during Gastrointestinal Endoscopy

**DOI:** 10.1371/journal.pone.0037614

**Published:** 2012-05-22

**Authors:** Yanhua Long, Hui-Hui Liu, Changhong Yu, Xia Tian, Yi-Ran Yang, Cheng Wang, Yajuan Pan

**Affiliations:** 1 Department of Gastroenterology, The Third Hospital of Wuhan (Tong Ren Hospital of Wuhan University), Wuhan, Hubei, People’s Republic of China; 2 Genomic Medicine Institute, Cleveland Clinic Foundation, Cleveland, Ohio, United States of America; 3 Quantitative Health Sciences, Cleveland Clinic Foundation, Cleveland, Ohio, United States of America; National Cancer Institute, United States of America

## Abstract

Hypoxemia is the most common adverse event that happened during gastrointestinal endoscopy. To estimate risk of hypoxemia prior to endoscopy, American Society of Anesthesiology (ASA) classification scores were used as a major predictive factor. But the accuracy of ASA scores for predicting hypoxemia incidence was doubted here, considering that the classification system ignores much information about general health status and fitness of patient that may contribute to hypoxemia. In this retrospective review of clinical data collected prospectively, the data on 4904 procedures were analyzed. The Pearson’s chi-square test or the Fisher exact test was employed to analyze variance of categorical factors. Continuous variables were statistically evaluated using t-tests or Analysis of variance (ANOVA). As a result, only 245 (5.0%) of the enrolled 4904 patients were found to present hypoxemia during endoscopy. Multivariable logistic regressions revealed that independent risk factors for hypoxemia include high BMI (BMI 30 versus 20, Odd ratio: 1.52, 95% CI: 1.13–2.05; P = 0.0098), hypertension (Odd ratio: 2.28, 95% CI: 1.44–3.60; P = 0.0004), diabetes (Odd ratio: 2.37, 95% CI: 1.30–4.34; P = 0.005), gastrointestinal diseases (Odd ratio: 1.77, 95% CI: 1.21–2.60; P = 0.0033), heart diseases (Odd ratio: 1.97, 95% CI: 1.06–3.68; P = 0.0325) and the procedures that combined esophagogastroduodenoscopy (EGD) and colonoscopy (Odd ratio: 4.84, 95% CI: 1.61–15.51; P = 0.0292; EGD as reference). It is noteworthy that ASA classification scores were not included as an independent predictive factor, and susceptibility of youth to hypoxemia during endoscopy was as high as old subjects. In conclusion, some certain pre-existing diseases of patients were newly identified as independent risk factors for hypoxemia during GI endoscopy. High ASA scores are a confounding predictive factor of pre-existing diseases. We thus recommend that youth (≤18 yrs), obese patients and those patients with hypertension, diabetes, heart diseases, or GI diseases should be monitored closely during sedation endoscopy.

## Introduction

Sedation was commonly used during endoscopy procedures because it can improve patient acceptance and tolerance via relieving discomfort and facilitating sympathetic patient management [1]. Lines of evidence demonstrated that sedation using optimal sedation and analgesia by endoscopist with or without help of anesthesiologist is safe and cost-efficient [1]–[3]. But cardiopulmonary adverse events (CAEs) that are related to conscious sedation occurred frequently during the procedures [1], [4]–[9].

Hypoxemia was intensively studied in past several years because of the incidences up to 1.5% to 70%, which make it the most common CAE during endoscopy [4], [8]–[15]. Hypoxemia can lead to many complications, depending on the severity of hypoxemia attack [15], [16]. When mild attacks occurred, patient may have headaches, restlessness or abnormal anxiety. In some cases, disorientation and confusion were observable. Severe hypoxemia may result in apnea, coma, hypotension, abnormal breathing pattern, and even myocardial ischemia during GI endoscopy [11], [17]–[19]. Hypoxemia may also cause discomfort of patients due to unsatisfactory sedation, even though it was transient and immediately corrected via oxygen supplementation [8], [14]. Therefore, identifying risk factors for hypoxemia is of clinical significance. So far its reported risk factors include high American Society of Anesthesiology (ASA) classification scores, old age of patient, high body mass index (BMI), conscious sedation and functional limitation of lung [4]–[9].

Here we re-estimated risk factors for hypoxemia because accuracy of ASA scores for predicting hypoxemia incidence was doubted. The current ASA classification divides patient’s status into only five or six categories. It is so vague that different grades were usually assigned to the same patient by different anesthesiologists [20]–[24], though some literatures suggested that the ASA grade of hypothetical patients can be accurately predicted by endoscopists [12], [25]. On the other hand, ASA classification ignores the information regarding patient’s pre-existing diseases, functional limitation and anxiety to clinical visit. Actually, many subjects had had pre-existing diseases or functional limitation before endoscopy procedures. These diseases or functional limitations, with exception of impaired lung function, may be potential independent risk factors for hypoxemia, but have never been studied. In this study we tested if pre-existing diseases of patient are independent risk factors for hypoxemia during endoscopy, and if high ASA scores are a confounding risk factor.

## Methods

### Objectives

The objectives of this study included **1**) to test if pre-existing diseases of patients are new independent risk factors for hypoxemia during GI endoscopy, and **2**) to test if ASA classification scores can predict risk of hypoxemia accurately.

### Ethics

This study was performed in strict accordance with the Helsinki Declaration. The protocol was discussed and approved by the Independent Ethics Committee (also called Institutional Review Board or IRB) of the Third Hospital of Wuhan. The subjects/patients were given sedation prior to endoscopy procedures by anesthesiologists or endoscopists, depending on patient’s choice, physical conditions and allergy to medication. Every effort was made to minimize suffering of endoscopy subjects. All enrolled subjects were required to sign consent form. If the subject was 18 year old or younger, the consent form was signed by the guardians of the participant. All data was analyzed anonymously.

### Eligibility Criteria

The endoscopy procedures were conducted from November 2004 to March 2010 in the Third Hospital of Wuhan (Wuhan, Hubei, P.R. China) by gastroenterologists who have experience of over 5,000 endoscopies, and the data analysis was accomplished in Cleveland Clinic Foundation (Cleveland, Ohio, USA). All patients in an age range from 15 year to 90 years and with ASA (American Society of Anesthesiology Classification) class I to IV were enrolled. Those patients who were unwilling or unable to sign consent form, or undergo the endoscopy procedures or emergency procedures, pregnant, patients with ASA V, and patients with pre-operative hypoxemia were excluded.

### Data Collection

Endoscopy subjects’ basic medical history and family history were collected, and physical examination was performed before endoscopy. Also recorded was the following information: name, race, age, gender, and weight, heights, history of smoking and alcohol intake, and type of endoscopy. ASA score was given to each patient prior to endoscopy. The pre-existing disease was diagnosed by primary doctor of patient. Blood pressure, heart rate, pulse oxygen saturation and use of medication were monitored continuously from the beginning of procedure to the end of recovery. Hypoxemia was defined as oxygen saturation less than 90% for at least 10 seconds during endoscopy and recovery period. The diagnosis of hypoxemia was confirmed via double checking if the sensor worked well.

### Sedative Dosages

Use and dose of sedative and analgesic medication were determined by the endoscopists performing the procedures, considering patient’s choice, physical conditions and allergy to medication. In this study 191 of the patients received no analgesic medication, propofol, and 574 of the patients received no sedative medication, midazolam. Generally, propofol of 1–100 mg or midazolam of 1–3 mg, if used, were initially given via vein based on patient’s physical conditions, such as age, weight, medical history including pre-existing disease, and feeling during the procedures. Additional doses were also used according to endoscopists’ evaluation of patient tolerance, with no limitation of the total dosage give. The complications related with sedation were diagnosed when the following events happened before endoscopy procedures: a SpO_2_<90%, systolic blood pressure less than 90 mmHg, diastolic pressure below 50 mmHg, heart rate <50 beats per min (bpm). Supplemental oxygen was offered to patient when oxygen desaturation decreased down to <90%. Physiological saline was used to adjust blood pressure when patient’s systolic blood pressure was <90 mmHg or the diastolic pressure was <50 mmHg. The resuscitation equipment and medications were always ready during the procedures and given to patient when needed.

### Statistics

The descriptive statistics including numbers and percentages of categorical variables, or mean and standard deviation of continuous variables were used to characterize the study cohort. The Pearson’s chi-square test was employed to analyze variance of categorical factors if patient number of a tested group was 5 or more. If patient number was less than 5, the Fisher exact test was used to evaluate statistical significance. Continuous variables were statistically evaluated using t-tests between two groups or Analysis of variance (ANOVA) among three or more groups. In this retrospective study, missing values in the risk factors were multiply imputed with 5 copies of imputed datasets before conducting statistical regression analysis in order to avoid potential selection bias if complete cases alone were used. Multivariable logistic regressions were performed independently with each of the 5 imputed datasets. After that, Rubin’s rule was used to aggregate the analysis results from all the 5 imputed datasets to construct a single logistic model for the CAE. All statistical inference and hypothetical tests were based on the final aggregated models. For numeric variables, restricted cubic splines were applied to accommodate the non-linear relationship with the outcomes [26]. A *p* value less than 0.05 was considered statistically significant. All analyses were performed with the open source statistical software R.12.2 (R Development Core Team, 2011) with MICE (Multivariate Imputation by Chained Equations) package added.

## Results

### Characteristics of Descriptive Statistics

A total of 4904 patients were enrolled in this study. All of them signed the consent form to participate. The statistical analysis was computed on these patients. As can be seen in Table 1 & 2, the study cohort was characterized by describing age, gender, body mass index (BMI), type of endoscopy, alcohol intake, allergy to medication, dose of midazolam, dose of propofol, ASA scores and pre-existing diseases. Only 245 (5.0%) of the patients presented hypoxemia during the endoscopy procedures with no statistically significant correlation with gender (Table 3 & 4).

**Table 1:** Overall statistical description for continuous variables (n = 4904).

Variables	Statistics
**Age**	58.1 (48,71)
**BMI**	23.5 (20.6, 26.1)
**Midazolam**	3.1 (1, 3)
**Propofol**	9.4 (5, 11)

*BMI* Body Mass Index.

Median (Q25, Q75) are presented in the table.

**Table 2:** Overall statistical description for categorical variables (n = 4904).

Variables	Sub-variables	Statistics
**Gender**	Male	2242 (45.7%)
	Female	2662 (54.3%)
**Alcohol**	Yes	1281 (26.1%)
	No	3619 (73.8%)
**Allergy**	Yes	259 (5.3%)
	No	3726 (76%)
**Endoscopy**	EGD	3902 (79.6%)
	Colonoscopy	980 (20%)
	EGD+colonoscopy	19 (0.4%)
**ASA scores**	I	1189 (24.2%)
	II	2364 (48.2%)
	III	756 (15.4%)
	IV	63 (1.3%)
**Lung disease**	No	4518 (92.5%)
	COPD	45 (0.9%)
	Chronic bronchitis	153 (3.1%)
	Pulmonary emphysema	27 (0.6%)
	Asthma	94 (1.9%)
	Others	45 (0.9%)
**Other preexisting diseases**	No	2103 (42.9%)
	Hypertension	615 (12.5%)
	Diabetes	165 (3.4%)
	CHF	108 (2.2%)
	Stroke	50 (1%)
	Liver cirrhosis	79 (1.6%)
	GI diseases	728 (14.8%)
	Cancer	68 (1.4%)
	Heart diseases	182 (3.7%)

*CHF* Chronic Heart Failure, *COPD* Chronic Obstructive Pulmonary Disease, *EGD* Esophagogastroduodenoscopy, *GI* gastrointestinal.

n (%) are presented in the table.

**Table 3:** Possible risk factors for hypoxemia during endoscopic procedures: Univariant analysis.

Variables (continuous)	No hypoxemia (n = 4659)	Hypoxemia (n = 245)	P value
**Age**	58±15.1	60.2±16.1	**0.0295**
**BMI**	23.4±4	24.4±4.5	**0.0006**
**Midazolam**	3.1±4.7	2.8±3.5	0.3628
**Propofol**	9.5±16.3	8.3±8.5	0.2469

*BMI* Body Mass Index.

Mean ± standard deviation are presented in the table.

**Table 4:** Possible risk factors for hypoxemia during endoscopic procedures: Univariant analysis (Continued).

Variables (categorical)	Subvariables	No hypoxemia (n = 4659)	Hypoxemia (n = 245)	P value
**Gender**				0.4292
	Male	2136 (45.8%)	106 (43.3%)	
	Female	2523 (54.2%)	139 (56.7%)	
**Alcohol**				0.3668
	No	3432 (73.7%)	187 (76.3%)	
	Yes	1223 (26.3%)	58 (23.7%)	
**Allergy**				**0.03498**
	No	3538 (93.7%)	188 (89.5%)	
	Yes	237 (6.3%)	22 (10.5%)	
**Endoscopy**				**0.03048**
	EGD	3708 (79.6%)	194 (79.2%)	
	Colonoscopy	933 (20%)	47 (19.2%)	
	EGD+colonoscopy	15 (0.3%)	4 (1.6%)	
**ASA scores**				**0.0005**
	I	1145 (27.7%)	44 (18.6%)	
	II	2238 (54.1%)	126 (53.2%)	
	III	693 (16.8%)	63 (26.6%)	
	IV	59 (1.4%)	4 (1.7%)	
**Lung diseases**				**0.0065**
	No	4306 (92.8%)	212 (86.9%)	
	COPD	40 (0.9%)	5 (2.0%)	
	Chronic bronchitis	141 (3.0%)	12 (4.9%)	
	Pulmonary emphysema	24 (0.5%)	3 (1.2%)	
	Asthma	84 (1.8%)	10 (4.1%)	
	Others	43 (0.9%)	2 (0.8%)	
**Other preexisting diseases**				**0.0005**
	No	2038 (52.3%)	65 (32.3%)	
	Hypertension	572 (14.7%)	43 (21.4%)	
	Diabetes	151 (3.9%)	14 (7.0%)	
	CHF	99 (2.5%)	9 (4.5%)	
	Stroke	47 (1.2%)	3 (1.5%)	
	Liver cirrhosis	73 (1.9%)	6 (3.0%)	
	GI diseases	683 (17.5%)	45 (22.4%)	
	Cancer	65 (1.7%)	3 (1.5%)	
	Heart diseases	169 (4.3%)	13 (6.5%)	

*CHF* Chronic Heart Failure, *COPD* Chronic Obstructive Pulmonary Disease, *EGD* Esophagogastroduodenoscopy, *GI* gastrointestinal.

n (%) are presented in the table.

### Hypoxemia Occurrence: Univariate Analysis

The results of univariate analysis were list in Table 3 and 4. Obviously, the incidence of hypoxemia has association with ASA scores, ages, BMI of patient, endoscopy types, pre-existing diseases and allergy to medication. Briefly, patients undergoing hypoxemia had higher ASA scores, older ages or higher BMI scores than those with no hypoxemia during endoscopy. Pre-existing diseases of patient contribute to hypoxemia significantly. Also, patients with history of allergy had relatively high susceptibility to hypoxemia, compared to others. But intake of alcohol, or medication for sedation did not alter incidence of hypoxemia during endoscopy. Still noticed was the trend that the less midazolam or propofol administrated, the higher incidence of hypoxemia (Table 3), but *p* values are higher than 0.05.

### Pre-existing Diseases but not ASA Scores as Risk Factor for Hypoxemia: Multivariate Analysis

In fact, many patients had had pre-existing diseases before receiving endoscopic examination or treatment. But the pre-existing diseases, with exception of impaired lung function, were not intensively studied as potential risk factor for the adverse events during endoscopy. To determine whether the pre-existing diseases are risk factors for hypoxemia, multivariate analysis was carried out on variables including gender, age, BMI, alcohol, allergy, endoscopy types, lung diseases, other pre-existing diseases, ASA scores, use of midzolam and propofol. The results in Table 5 & 6 showed that based on *p* value less than 0.05, BMI, endoscopy types, pre-existing diseases were the independent risk factors for hypoxemia.

**Table 5:** Independent risk factors for hypoxemia: Multivariant analysis.

Variable (continuous)	Subvariable	Odd ratio	95% CI	P value
**Age**		0.94	0.73–1.21	0.1365
**BMI**				**0.0098**
	<20	0.94	0.63–1.06	
	≥20	1.52	1.13–2.06	
**Midazolam**		0.83	0.65–1.06	0.196
**Propofol**		0.84	0.67–1.06	0.2266

*BMI* Body Mass Index.

**Table 6:** Independent risk factors for hypoxemia: Multivariant analysis (Continued).

Variables (categorical)		Subvariables	Odd ratio		95% CI	P value
**Gender**						0.6887
		Male	1		Reference	
		Female	1.06		0.80–1.42	
**Alcohol**						0.5023
		No	1		Reference	
		Yes	0.89		0.63–1.25	
**Allergy**						0.2923
		No	1		Reference	
		Yes	1.28		0.81–2.04	
**Endoscopy**						0.0292
		EGD		1	Reference	
		Colonoscopy		1.04	0.75–1.46	
		EGD+colonoscopy		4.84	1.51–15.5	
**ASA scores**						0.5723
		I		1	Reference	
		II		1.13	0.76–1.67	
		III		1.4	0.85–2.29	
		IV		1.21	0.40–3.69	
**Lung diseases**						0.1499
		No		1	Reference	
		COPD		2.02	0.75–5.47	
		Chronic bronchitis		1.65	0.88–3.08	
		Pulmonary emphysema		2.11	0.59–7.51	
		Asthma		2	0.99–4.02	
		Others		1.18	0.28–5.04	
**Other preexisting diseases**						**0.0094**
		No		1	Reference	
		Hypertension		2.28	1.44–3.60	0.0004
		Diabetes		2.37	1.29–4.34	0.005
		CHF		1.88	0.81–4.34	0.1407
		Stroke		2.07	0.76–5.67	0.1557
		Liver cirrhosis		1.70	0.69–4.18	0.2459
		GI diseases		1.77	1.21–2.60	0.0033
		Cancer		1.58	0.48–5.11	0.4494
		Heart diseases		1.97	1.06–3.68	0.0325

*CHF* Chronic Heart Failure, *COPD* Chronic Obstructive Pulmonary Disease, *EGD* Esophagogastroduodenoscopy, *GI* gastrointestinal.

**Figure 1:**
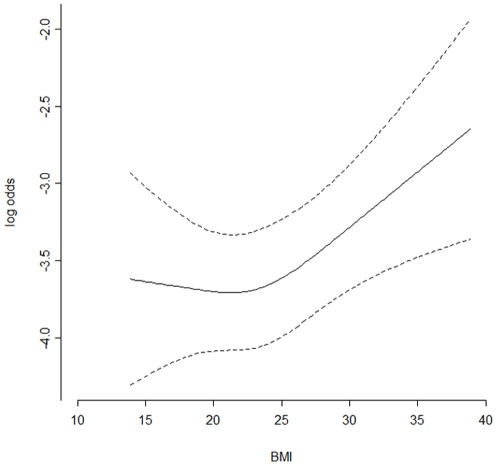
The relation between odd ratio of hypoxemia and body mass index.

In detail, **1**) the relation between incidence of hypoxemia and BMI is non-linear (Figure 1). If BMI is 20 or less, odd ratio of hypoxemia during GI endoscopy kept nearly constant, and did not vary with BMI. But when BMI value exceeds 20, the higher BMI value, the higher odd ratio of hypoxemia (BMI 30 versus 20: OR: 1.52, 95% CIs: 1.13–2.05, P = 0.0098). **2**) Some pre-existing diseases were newly identified as independent risk factors for hypoxemia. For example, risk of hypoxemia during GI endoscopy was increased when the subjects of endoscopy had hypertension (Odd ratio: 2.28 95% CI: 1.44–3.60; P = 0.0004). Hypoxemia was also relatively common in the patients with diabetes, versus the control group, in which subjects had no pre-existing disease (Odd ratio: 2.37, 95% CI: 1.29–4.34; P = 0.005). The patients with gastrointestinal diseases presented high susceptibility to hypoxemia (Odd ratio: 1.77, 95% CI: 1.21–2.60; P = 0.0033). Moreover, hypoxemia was found to develop in 13 (7.14%) of 182 patients with heart diseases (Odd ratio: 1.97, 95% CI: 1.06–3.68; P = 0.0325, Table 6), but among 2103 subjects with no pre-existing disease, only 65 (3.09%) had hypoxemia during the procedures. It suggested that heart diseases increase susceptibility of patient to hypoxemia (Table 4). **3**) In addition to high BMI and pre-existing diseases, the procedures that combined EGD and colonoscopy may also increase incidence of hypoxemia, likely because this combined procedure generally took longer duration than other procedures. **4**) Interestingly, the results obtained in this study showed that high ASA scores did not contribute to hypoxemia incidence, which is contrary to previous studies (Table 6).

## Discussion

Hypoxemia is the most common cardiopulmonary adverse events (CAEs) that may cause morbidity and mortality during endoscopy procedures [8], [12], [13]. The reported risk factors for hypoxemia included high ASA scores, conscious sedation, obesity, old age of patient and function limitation of lung [4]–[9], [11]–[14]. In this study we re-estimated risk factors for hypoxemia, and newly identified hypertension, diabetes, GI diseases, and heart diseases as its independent risk factors. Interestingly, high ASA scores were not included as an independent risk factor for hypoxemia in this study.

ASA scores have been used to predict risks of CAEs during GI endoscopy for several decades. It has been indicated that incidences of CAEs increase with ascending ASA class. Compared to ASA I and ASA II, ASA III had a 1.8-fold increase in odd ratio of CAEs, the correspondingly increased fold of ASA V patients was 3.2, and ASA V was 7.5 [12]. The relative risks of ASA III/IV/V patients to develop hypoxemia also doubled the risks of ASA I/II patients [13]. But on the other side, some anesthesiological literatures reported that ASA classification was so vague that different grades were usually assigned to the same patient by different anesthesiologists [20]–[24]. These made us doubt appropriateness of ASA classification scores for predicting risks of CAEs, e.g. hypoxemia.

To further validate our finding that high ASA scores are not a real risk factor for hypoxemia, we excluded the pre-existing diseases from the list of risk factors of hypoxemia and re-analyzed the data statistically. As expected, high ASA scores showed significant effects on increasing susceptibility to hypoxemia when pre-existing diseases were excluded (Table 7). The results thus demonstrated that high ASA scores are a confounding risk factor of pre-existing diseases for hypoxemia during endoscopy. In other words, ASA classification distorted the observed association between hypoxemia and some pre-existing diseases.

**Table 7:** Independent risk factors for hypoxemia with excluding pre-existing diseases.

Variables	Subvariable	Odd ratio	95% CI	P value
**Age**		0.99	0.78–1.26	0.1716
**BMI**		1.24	1.03–1.50	**0.0017**
**Midazolam**		0.84	0.65–1.08	0.2140
**Propofol**		0.84	0.66–1.06	0.2545
				
**Gender**				0.8503
	Male	1	Reference	
	Female	1.03	0.77–1.37	
**Alcohol**				0.5877
	No	1	Reference	
	Yes	0.91	0.65–1.28	
**Allergy**				0.1480
	No	1	Reference	
	Yes	1.41	0.88–2.26	
**Endoscopy**				**0.0331**
	EGD	1	Reference	
	Colonoscopy	0.98	0.71–1.37	
	EGD+colonoscopy	4.56	1.45–14.33	
**ASA scores**				**0.0419**
	I	1	Reference	
	II	1.38	0.94–2.02	
	III	1.95	1.23–3.10	
	IV	1.51	0.51–4.50	
**Lung diseases**				0.3699
	No	1	Reference	
	COPD	1.68	0.63–4.48	
	Chronic bronchitis	1.48	0.79–2.76	
	Pulmonary emphysema	1.84	0.53–6.42	
	Asthma	1.79	0.89–3.59	
	Others	0.95	0.22–4.06	

*BMI* Body Mass Index, *CHF* Chronic Heart Failure, *COPD* Chronic Obstructive Pulmonary Disease, *EGD* Esophagogastroduodenoscopy.

In order to determine the association between pre-existing diseases and incidence of hypoxemia, we chose 2,103 subjects with no pre-existing disease as control group. The pre-existing diseases studied here included hypertension, diabetes, CHF, history of stroke, liver cirrhosis, cancer, heart disease, gastrointestinal diseases, and some lung diseases, such as COPD, chronic bronchitis, pulmonary emphysema, and asthma. These diseases frequently occurred in developed and developing countries. As can be seen in Table 4, hypoxemia developed commonly in the patients with hypertension, diabetes, heart diseases, or gastrointestinal diseases, and those with COPD, asthma, or pulmonary emphysema. But of these studied diseases, only hypertension, diabetes, GI diseases and heart diseases contributed to incidence of hypoxemia independently, based on the results from multivariable logistic regressions (Table 6).

The possible mechanism of pre-existing diseases increasing hypoxemia risk may be various. For example, **1**) it has been demonstrated that chronic exposure to intermittent hypoxemia can cause hypertension. The mechanism involved sympathetic nervous system overactivity, oxidative stress and endothelial dysfunction [27], [28]. In our study cohort, cause of hypertension remained unclear. But it is possible that those patients with hypertension were intermittently hypoxemic prior to endoscopy, and thus became more susceptible to hypoxemia than others. **2**) Diabetes mellitus is another pre-existing disease that contributed to hypoxemia during endoscopy. It was reported that diabetes can adversely affect breath to increase risk for severe nocturnal hypoxemia [29]. The same mechanism may be employed to explain our finding that hypoxemia happened commonly in the patients with diabetes. Moreover, in some cases, diabetes turned blood acidic, which decreased affinity of hemoglobin to oxygen. The oxygen saturation was thus reduced in these patients. **3**) Of the patients with gastrointestinal diseases, a majority underwent therapeutic procedures, which took relatively long duration. Therefore, the patients with the GI diseases had more chance to be hit by hypoxemia during GI endoscopy. **4**) In the patients with heart diseases, we noticed that the average value of preoperative oxygen saturation was 97.66%, which is significantly smaller than that (98.74%) of the endoscopy subjects with no pre-existing disease (p<0.00001). That is, the patients with heart diseases had been relatively hypoxemic before endoscopic procedures, compared with the control group, though their oxygen saturation was still in a normal range. This may be due to reduced cardiac output resulting from the heart diseases. Consequently, the tolerance of these patients with heart diseases to hypoxemia was thus decreased during endoscopy. Although some explanations on the association between pre-existing diseases and hypoxemia were given here, the mechanisms still need to be confirmed further.

In this study, we found that the direction of the associations between risk of hypoxemia and the dose of midazolam and propofol was opposite to the conclusion that incidence of hypoxemia increases with the dose of midazolam and propofol. But those associations were not statistically significant with p-values >0.05. In the baseline table, the unadjusted statistics also showed that the mean midazolam or propofol is lower in patients with hypoxemia, but again this difference is not statistically significant. That is, there seems some indication that midazolam or propofol has lower dose for patients with hypoxemia, but our data doesn’t support this with enough statistical significance. Actually, before the endoscopy procedures all endoscopists enrolled in this study had already known that analgesics and sedatives may contribute to occurrence of hypoxemia. The endoscopists administered low lose to those who have high risk of hypoxemia, especially when hypoxemia happened. As the result, those who are susceptible to hypoxemia were administered lower dose of sedation and analgesia than others. We also noticed that in a well design prospective study, high dose of medication may cause high risk of hypoxemia [8]. But in other studies, doses of sedation and analgesia were not identified as risk factor for hypoxemia [6]. It was even reported that Midazolam helps decrease incidence of cardiopulmonary events during endoscopy procedures (OR: 0.93; 95% CI: 0.91–0.95) [12]. Moreover, Propofol sedation during colonoscopy appears to have lower odds of cardiopulmonary complications compared with other traditional agents [7]. The reason may be various. In our data, it is obvious that high risk of hypoxemia forced the endoscopists to administer low dose rather than low dose of medication caused high risk of hypoxemia.

**Figure 2:**
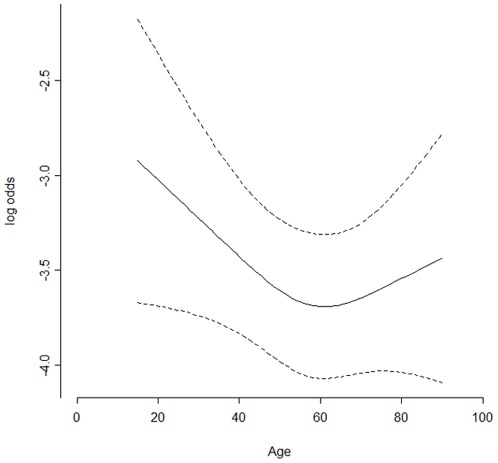
The relation between odd ratio of hypoxemia and patient age.

Old age of patient is another controversial risk factor for hypoxemia. It increased incidence of this adverse event in some cases [8], [10], [13], but was not included as independent risk factor in our study and another one [16]. These might be due to different population, definition of hypoxemia, and statistical analysis. In our study, all enrolled patients were in a range from 15 years to 90 years. Our range of age was wider than other studies. Moreover, we did not categorize the patients according to age. Namely, age of patient was analyzed as a continuous variable. As a result, we found that the relation between hypoxemia and patient age was non-linear, and hypoxemia developed in young subjects (≤18 years) as commonly as in older subjects (Figure 2). A possible explanation is that anxiety may be more common and stronger in the youth during clinic visit than in adult. It likely made young subjects relatively susceptible to hypoxemia when undergoing endoscopy. Although the explanation needs to be further verified, it still suggested that we should put more attention on monitoring health status of young subjects and occurrence of hypoxemia during GI endoscopy.

### Limitations

Here we retrospectively analyzed the clinical data that were prospectively collected. The study thus has some limitations. **1**) The endoscopic procedures were performed in only one hospital. The patient population and the gastroenterologists operating endoscope may not be representative. However, we noticed that many results obtained in this study are consistent with previous reports [8], [12], [13], [16], [30]. For example, there were no difference in hypoxemia incidence between male patients and females. Intake of alcohol had no effect on hypoxemia occurrence, but old age and high BMI increase risk of hypoxemia [8]. These suggested that our results may be generalizable. **2**) The data were collected and entered by gastroenterologists and nurses who were not trained on the standard clinical data entry. Some information about patient conditions or endoscopy procedures was missed (Table S1). We thus imputed every missing 5 copies to minimize the bias, based on an accepted strategy of statistics [26], [31]. As can be seen, our data led to many conclusions that were consistent with previous literatures, when the pre-existing diseases were not estimated as potential risk factors for the hypoxemia. Hence we believed that our data can reflect the truth with no significant bias with other studies. **3**) For some potential risk factors, the sample size was too small to obtain convincible results of statistics. It may increase possibility of getting false negative results. Fortunately, we had enough case numbers to show the effects of pre-existing diseases on incidence of hypoxemia, and identify high ASA scores as a confounding predictive factor of pre-existing diseases. Therefore the results we showed here may be convinced. **4**) The pre-existing diseases estimated in this study only included some ‘systemic’ diseases, i.g. hypertension, diabetes, and some ‘local’ diseases i.g. heart diseases, GI diseases, liver cirrhosis and so on. So far we do not exclude that some other pre-existing diseases could be potential risk factors for hypoxemia during GI endoscopy. **5**) Some pre-existing diseases that we discussed here were not well defined. For example, ‘GI diseases’ include esophagitis, gastritis, cholelithiasis complicated by bile reflux gastritis, gastric ulcer, duodenum ulcer, and proctocolitis. Heart diseases are a variety of diseases affecting heart, mainly including coronary heart disease, ischaemic heart diseases, cardiomyopathy and arrhythmia. We did not determine effects on hypoxemia of each kind of GI diseases and heart diseases. But we still believe that this study had shown new angle of view for association of risk factors and hypoxemia that occurred during GI endoscopy, and will provide useful information to clinically prevent these adverse events.

## Conclusions

In conclusion, some pre-existing diseases of patients who underwent endoscopic procedures may increase risk of hypoxemia. High ASA scores are a confounding risk factor of pre-existing diseases for hypoxemia. We recommend that youth, obese patients and those patients with hypertension, diabetes, heart diseases and GI diseases should be monitored closely during sedation endoscopy.

## Supporting Information

Table S1 **Number of missing** ***BMI*** **Body Mass Index.**
(DOC)
