# Supplementary material for: Pre-Existing Diseases of Patients Increase Susceptibility to Hypoxemia during Gastrointestinal Endoscopy
Source: PLoS One. 2012 May 22;7(5):e37614. doi: 10.1371/journal.pone.0037614 (PMC3358262; doi:10.1371/journal.pone.0037614)
Supplement: Table S1 — Number of missing BMI Body Mass Index. (DOC) [file pone.0037614.s001.doc]

| Supplementary table S1: Number of missing   | **Variables** | **No hypoxemia** | **Hypoxemia** | | --- | --- | --- | | **Age** | 90 | 1 | | **Gender** | 0 | 0 | | **Alcohol** | 4 | 0 | | **Allergy** | 884 | 35 | | **Endoscopy** | 3 | 0 | | **ASA scores** | 524 | 8 | | **Lung diseases** | 21 | 1 | | **Other preexisting diseases** | 762 | 44 | | **BMI** | 133 | 3 | | **Midazolam** | 113 | 3 | | **Propofol** | 60 | 4 |   *BMI* Body Mass Index |
| --- | --- | --- | --- | --- | --- | --- | --- | --- | --- | --- | --- | --- | --- | --- | --- | --- | --- | --- | --- | --- | --- | --- | --- | --- | --- | --- | --- | --- | --- | --- | --- | --- | --- | --- | --- | --- |
